# Supplementary material for: HLA class I peptide polymorphisms contribute to class II DQβ0603:DQα0103 antibody specificity
Source: Nat Commun. 2024 Jan 19;15:609. doi: 10.1038/s41467-024-44912-0 (PMC10798988; doi:10.1038/s41467-024-44912-0)
Supplement: Supplementary file 1 — supplementary information [file 41467_2024_44912_MOESM1_ESM.pdf]

HLA class I peptide polymorphisms contribute to class II DQβ0603:DQα0103 antibody specificity

N. Remi Shih[1,&], Thoa Nong[1,&], Cathi Murphey[2], Mayra Lopez-Cepero[1], Peter W. Nickerson[3], Jean-luc Taupin[4], Magali Devriese[4], Jakob Nilsson[5], Marie-Benedicte Matignon[6], Robert A. Bray[7,#] and Jar-How Lee[1,#,*]

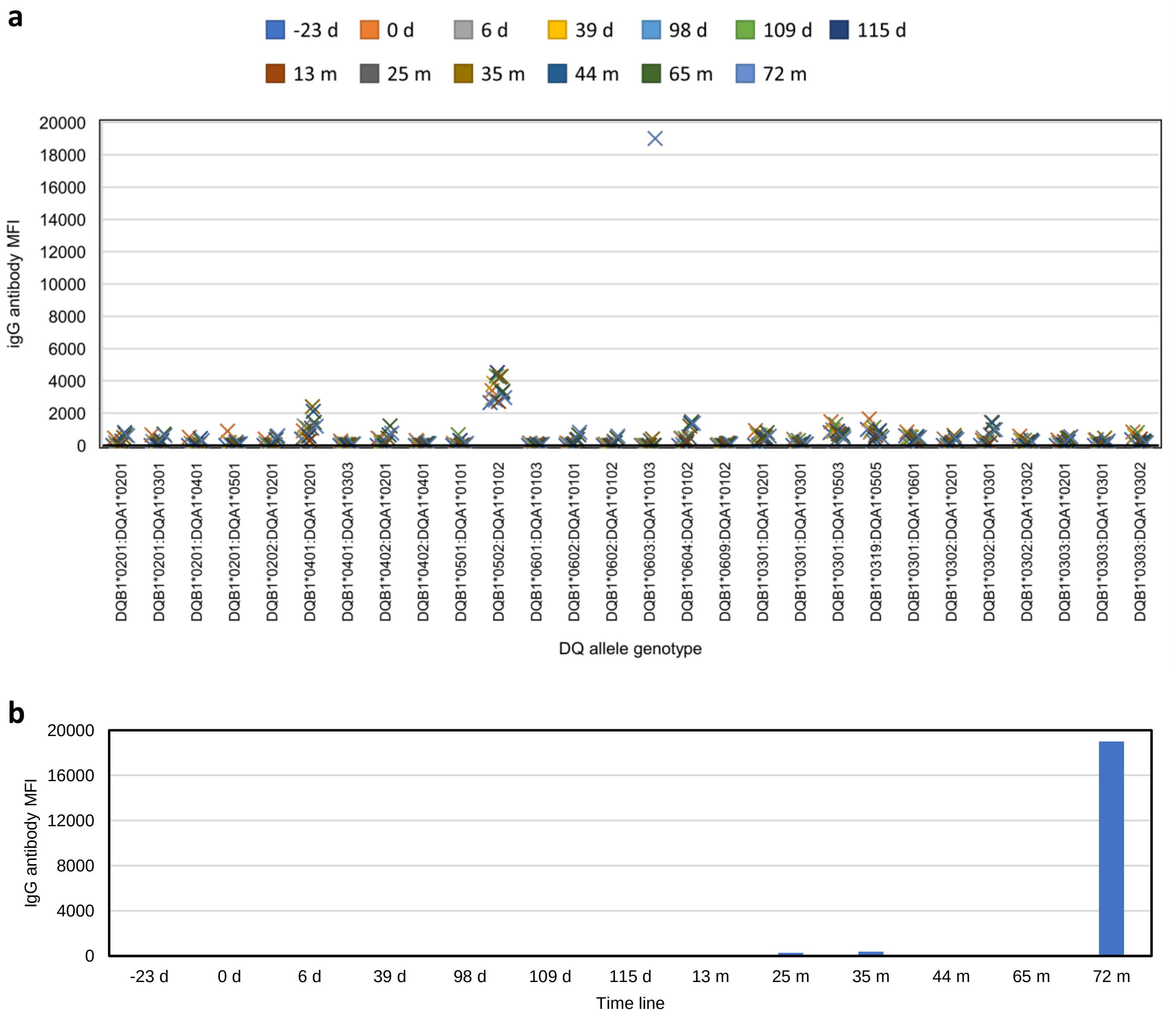

Supplementary Figure 1. DQ antibody profiles tracking of a patient, S22, receiving DQβ0603:DQα0103 mismatch donor kidney. DQ antibody profiles from -23 days (-23 d) prior to transplant to 72 months (72 m) post transplant were generated from the results of the LABScreen class II SAB assay (One Lambda Inc., West Hill, CA). a. A total of thirteen tests were performed with shorter intervals in the first 4 months and yearly afterward. b. the reactivity of DQβ0603:DQα0103 beads were plotted separately to identify the time that DQβ0603:DQα0103 antibody appeared.

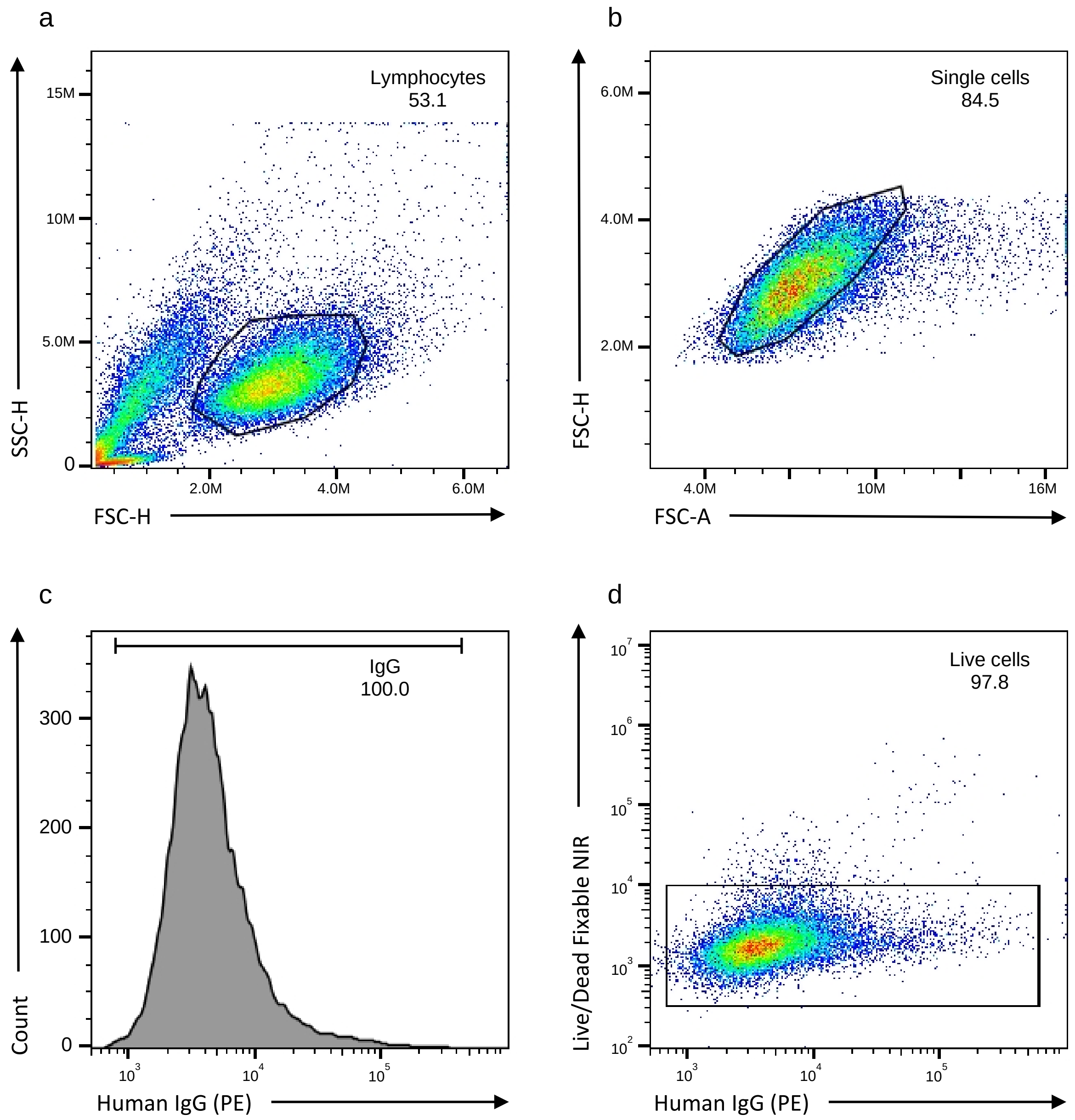

Supplementary Figure 2. Flow analysis gating strategy. Using the LCLKO DQβ06:03DQα01:03 transfectant stained with sera S1 as an example. See methods for cell staining and labeling details. Cells were first gated for lymphocyte population (a) and then single cells (b). Live cells (d, labelled by Live/Fixable NIR from Thermofisher Scientific, L34975) were gated before histograms (c) were applied.

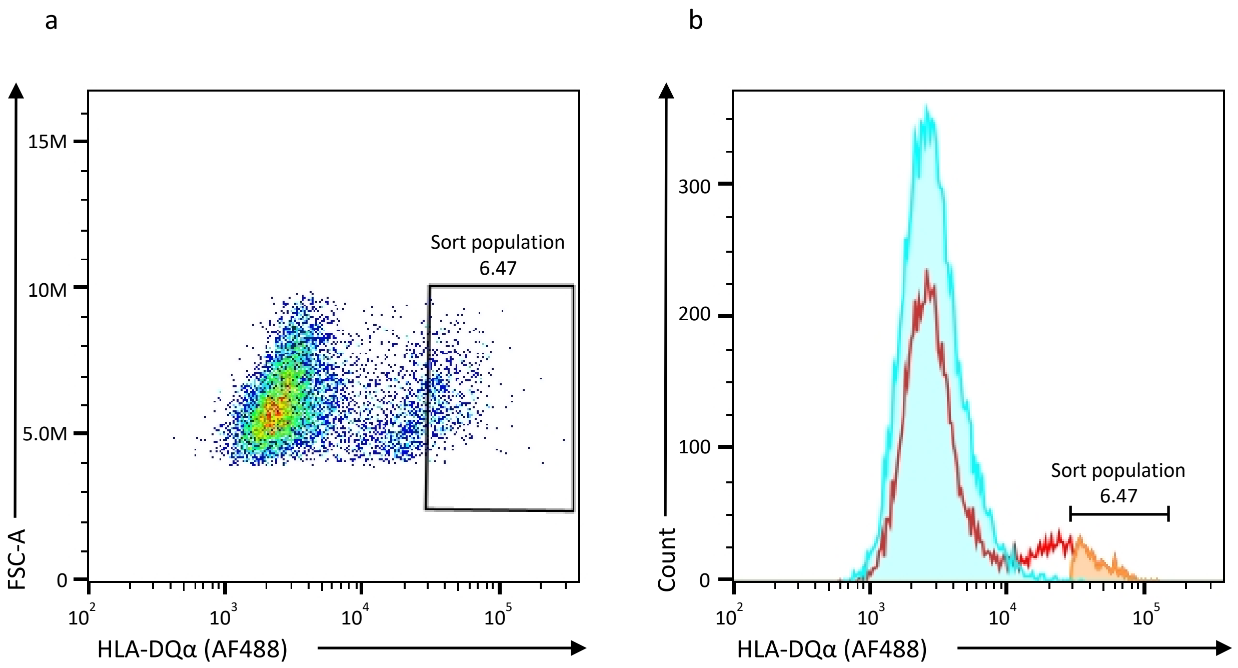

Supplementary Figure 3. Sorting Strategies on DQβ0603:DQα0103 transfectants.

Blasticidin resistant transfectants (T2 transfected by plasmids encoding DQB1*06:03 and DQA1*01:03) were labelled with Alexa Fluor® FM5148 (1 µg per 1 million cells in 100 µl cell wash buffer) and loaded into Sony MA900, see methods for detail. a. Box gate on the area where high immunofluorescent cells were collected. b. The corresponding box gate (orange) over the T2 transfectant histogram (red) indicated the location of high immunofluorescent cells. Un-transfected T2 host (blue) is used to establish the baseline.

Supplementary Table 1. Peptide binding efficiency of DQβ0603:DQα0103protein from different transfectant hosts.

	K562	BLS	T2	T2DM	LCLKO	LCL3023
A1	2055	4649	3749	1233	1987	1436
A2	1339	4278	3395	690	1236	918
A3	3105	6081	4846	1221	2169	1189
B1	2568	4389	3719	1421	2197	1404
B2	5108	6920	6426	3851	4850	2962
B3	1276	3543	2851	656	1292	878
C1	2154	4732	4126	1184	1937	1421
C2	2263	4560	4110	1301	2063	1534
E1	1301	4123	3283	494	1106	737
F1	1823	4730	3626	886	1575	1023
DRA1	1466	4755	3689	779	1300	1067

Peptide binding efficiency was measured by the SAPE reactivity of the biotinylated peptides following the peptide loading protocol.

Supplementary Table 2. Patient DQ typing

Serum ID	DQB1	DQB1	DQA1	DQA1
S1	03:02	03:02	NA	NA
S2	03:19	04:02	05:05	04:01
S3	03:01	03:02	03:03	05:05
S4	03:02	05:01	01:01	03:01
S5	03:02	06:02	01:02	03:01
S6	04:02	04:02	04:01	04:01
S7	5	7	NA	NA
S8	02:02	03:01	02:01	03:03
S9	03:01	05:01	01:01	05:05
S10	2	7	NA	NA
S11	03:02	05:03	01:01	03:01
S12	03:01	03:01	03:03	05:05
S13	02:02	03:01	02:01	03:03
S14	5	8	01	03
S15	03:01	03:01	03:03	03:03
S16	03:01	06:02	01:02	03:01
S17	2	5	NA	NA
S18	02:02	06:04	01:02	02:01
S19	02:02	06:02	01:02	02:01
S20	02:02	05:01	01:01	02:01
S21	2	5	NA	NA
S22	06:09	04:02	01:02	04:01

NA: not available
