# Supplementary material for: HLA class I peptide polymorphisms contribute to class II DQβ0603:DQα0103 antibody specificity
Source: Nat Commun. 2024 Jan 19;15:609. doi: 10.1038/s41467-024-44912-0 (PMC10798988; doi:10.1038/s41467-024-44912-0)
Supplement: Supplementary file 3 — Reporting Summary [file 41467_2024_44912_MOESM3_ESM.pdf]

nature portfolio

Corresponding author(s): Jar-How Lee

Last updated by author(s): Dec 23, 2023

Reporting Summary

Nature Portfolio wishes to improve the reproducibility of the work that we publish. This form provides structure for consistency and transparency in reporting. For further information on Nature Portfolio policies, see our Editorial Policies and the Editorial Policy Checklist.

Statistics

For all statistical analyses, confirm that the following items are present in the figure legend, table legend, main text, or Methods section.

n/a	Confirmed	
☐	☒	The exact sample size (*n*) for each experimental group/condition, given as a discrete number and unit of measurement
☐	☒	A statement on whether measurements were taken from distinct samples or whether the same sample was measured repeatedly
☐	☒	The statistical test(s) used AND whether they are one- or two-sided<br>*Only common tests should be described solely by name; describe more complex techniques in the Methods section.*
☐	☒	A description of all covariates tested
☐	☒	A description of any assumptions or corrections, such as tests of normality and adjustment for multiple comparisons
☐	☒	A full description of the statistical parameters including central tendency (e.g. means) or other basic estimates (e.g. regression coefficient) AND variation (e.g. standard deviation) or associated estimates of uncertainty (e.g. confidence intervals)
☐	☒	For null hypothesis testing, the test statistic (e.g. *F*, *t*, *r*) with confidence intervals, effect sizes, degrees of freedom and *P* value noted<br>*Give P values as exact values whenever suitable.*
☒	☐	For Bayesian analysis, information on the choice of priors and Markov chain Monte Carlo settings
☒	☐	For hierarchical and complex designs, identification of the appropriate level for tests and full reporting of outcomes
☒	☐	Estimates of effect sizes (e.g. Cohen's *d*, Pearson's *r*), indicating how they were calculated

Our web collection on statistics for biologists contains articles on many of the points above.

Software and code

Policy information about availability of computer code

Data collection	Luminex xPonent for FLEXMAP3D Version 4.3 Update 1 Build 309 was used for data collection
Data analysis	Statistical significance was determined using one-way analysis of variance (ANOVA) followed by Bonferroni post hoc test or using Student's t test in Microsoft® Excel® for Microsoft 365 MSO (Version 2311 Build 16.0.17029.20028)

For manuscripts utilizing custom algorithms or software that are central to the research but not yet described in published literature, software must be made available to editors and reviewers. We strongly encourage code deposition in a community repository (e.g. GitHub). See the Nature Portfolio guidelines for submitting code & software for further information.

Data

Policy information about availability of data

All manuscripts must include a data availability statement. This statement should provide the following information, where applicable:
- Accession codes, unique identifiers, or web links for publicly available datasets
- A description of any restrictions on data availability
- For clinical datasets or third party data, please ensure that the statement adheres to our policy

The mass spectrometry proteomics data have been deposited to the ProteomeXchange Consortium via the PRIDE partner repository with the dataset identifier PXD043999 (http://www.ebi.ac.uk/pride/archive/projects/PXD043999)

Research involving human participants, their data, or biological material

Policy information about studies with human participants or human data. See also policy information about sex, gender (identity/presentation), and sexual orientation and race, ethnicity and racism.

Reporting on sex and gender	No human subject was used in this study
Reporting on race, ethnicity, or other socially relevant groupings	No human subject was used in this study
Population characteristics	No human subject was used in this study
Recruitment	No human subject was used in this study
Ethics oversight	No human subject was used in this study

Note that full information on the approval of the study protocol must also be provided in the manuscript.

Field-specific reporting

Please select the one below that is the best fit for your research. If you are not sure, read the appropriate sections before making your selection.

[x] Life sciences   [ ] Behavioural & social sciences   [ ] Ecological, evolutionary & environmental sciences

For a reference copy of the document with all sections, see nature.com/documents/nr-reporting-summary-flat.pdf

Life sciences study design

All studies must disclose on these points even when the disclosure is negative.

Sample size	Sample size was determined based on the availability of biological materials
Data exclusions	No data was excluded
Replication	All experiments were repeated 3 times (n=3) except for the experiments in Figure 5 which were done in duplicate (n=2).
Randomization	Randomization is not relevant for this study since all serum samples were selected based on specific antibody reactivity.
Blinding	Blinding is not relevant for this study since all serum samples were selected based on specific antibody reactivity.

Reporting for specific materials, systems and methods

We require information from authors about some types of materials, experimental systems and methods used in many studies. Here, indicate whether each material, system or method listed is relevant to your study. If you are not sure if a list item applies to your research, read the appropriate section before selecting a response.

Materials & experimental systems

n/a	Involved in the study	
[ ]	[x]	Antibodies
[ ]	[x]	Eukaryotic cell lines
[x]	[ ]	Palaeontology and archaeology
[x]	[ ]	Animals and other organisms
[x]	[ ]	Clinical data
[x]	[ ]	Dual use research of concern
[x]	[ ]	Plants

Methods

n/a	Involved in the study	
[x]	[ ]	ChIP-seq
[ ]	[x]	Flow cytometry
[x]	[ ]	MRI-based neuroimaging

Antibodies

Antibodies used	DQ1 antibody Genox 3.53 (Thermo Fisher Scientific, Catalog # MA1-46297DQ1)<br>DQ1,3 antibody HL-37 (Thermo Fisher Scientific, Cat #MA1-19143DQ1,3)<br>Mouse IgG isotype control MOPC-21 (Thermo Fisher Scientific, Cat # MA1-10407)<br>DQ antibody Tu169 (BioLegend, Cat #361502)<br>R-Phycoerythrin labeled Goat Anti-Mouse IgG secondary antibody(Jackson ImmunoResearch Laboratories, cat #115-116-146)<br>R-Phycoerythrin labeled Goat Anti-Human IgG secondary antibody (Jackson ImmunoResearch Laboratories, (cat #115-116-146)

	FM5148 and FR3315 were provided by One Lambda Inc. under a Material Transfer Agreement.
Validation	All antibodies were validated by the manufacturer and data is available at the manufacturer's website as indicated below:<br>HL-37 (https://www.thermofisher.com/antibody/product/HLA-DQ1-DQ3-Antibody-clone-HL-37-Monoclonal/MA1-19143)<br>Genox 3.53 (https://www.thermofisher.com/antibody/product/HLA-DQw1-Antibody-clone-Genox-3-53-Monoclonal/MA1-46297)<br>Tu169 (https://www.biolegend.com/ja-jp/products/purified-anti-human-hla-dq-antibody-9334)<br>MOPC-21 (https://www.thermofisher.com/antibody/product/Mouse-IgG1-clone-MOPC-21-Isotype-Control/MA1-10407)<br>R-Phycoerythrin labeled Goat Anti-Mouse IgG secondary antibody(https://www.jacksonimmuno.com/catalog/products/115-116-146)<br>R-Phycoerythrin labeled Goat Anti-Human IgG secondary antibody (https://www.jacksonimmuno.com/catalog/products/109-116-098)

Eukaryotic cell lines

Policy information about cell lines and Sex and Gender in Research

Cell line source(s)	9058, 9060, 9062, 9065 and 9105 can be purchased from International Histocompatibility Working Group (https://www.fredhutch.org/en/research/institutes-networks-ircs/international-histocompatibility-working-group.html)<br>K562 and T2 cell lines were purchased from ATCC.<br>LCL3023 was a gift from Dr. William Burlingham, University of Wisconsin – Madison, USA.<br>BLS B cell line was a gift from Dr. Roland Martin, University of Zurich, Switzerland.<br>LCLKO and T2DM were provided by One Lambda Inc. under a Material Transfer Agreement.
Authentication	HLA typing of all cell lines were confirmed by next generation sequencing.
Mycoplasma contamination	All cells were tested negative for mycoplasma contamination.
Commonly misidentified lines (See ICLAC register)	No commonly misidentified lines were used in this study.

Flow Cytometry

Plots

Confirm that:

- [x] The axis labels state the marker and fluorochrome used (e.g. CD4-FITC).
- [x] The axis scales are clearly visible. Include numbers along axes only for bottom left plot of group (a 'group' is an analysis of identical markers).
- [ ] All plots are contour plots with outliers or pseudocolor plots.
- [ ] A numerical value for number of cells or percentage (with statistics) is provided.

Methodology

Sample preparation	Approximately 10e6cells from the stable transfectants were mixed with 50 µl of serum and incubated for 20 minutes at 4°C. After incubation, cells were washed with 1X PBS, 0.1% glucose and then incubated with biotinylated F(ab')2-Goat anti-Human IgG Fc-specific antibody (Invitrogen) for 20 minutes. Samples were washed and incubated with R-Phycoerythrin Streptavidin (Jackson ImmunoResearch Laboratories, West Grove, PA). After washing, samples were acquired on a flow cytometer
Instrument	Quanteon Flow Cytometer (Agilent Technologies, Santa Clara, CA).
Software	Data was analyzed with FlowJo Software (https://www.flowjo.com/)
Cell population abundance	Transfectants of EBV transformed B cell lines
Gating strategy	Live cell gating

- [x] Tick this box to confirm that a figure exemplifying the gating strategy is provided in the Supplementary Information.
